# 5-HT7 receptor activation promotes an increase in TrkB receptor expression and phosphorylation

**DOI:** 10.3389/fnbeh.2014.00391

**Published:** 2014-11-07

**Authors:** Anshula Samarajeewa, Lolita Goldemann, Maryam S. Vasefi, Nawaz Ahmed, Nyasha Gondora, Chandni Khanderia, John G. Mielke, Michael A. Beazely

**Affiliations:** ^1^School of Pharmacy, University of WaterlooKitchener, ON, Canada; ^2^Department of Pharmacy, University of BaselBasel, Switzerland; ^3^School of Pharmacy, University College LondonLondon, UK; ^4^School of Public Health and Health Systems, University of WaterlooWaterloo, ON, Canada

**Keywords:** 5-HT7, TrkB, transactivation, phosphorylation, protein expression

## Abstract

The serotonin (5-HT) type 7 receptor is expressed throughout the CNS including the cortex and hippocampus. We have previously demonstrated that the application of 5-HT7 receptor agonists to primary hippocampal neurons and SH-SY5Y cells increases platelet-derived growth factor (PDGF) receptor expression and promotes neuroprotection against N-methyl-D-aspartate-(NMDA)-induced toxicity. The tropomyosin-related kinase B (TrkB) receptor is one of the receptors for brain-derived neurotrophic factor (BDNF) and is associated with neurodevelopmental and neuroprotective effects. Application of LP 12 to primary cerebral cortical cultures, SH-SY5Y cells, as well as the retinal ganglion cell line, RGC-5, increased both the expression of full length TrkB as well as its basal phosphorylation state at tyrosine 816. The increase in TrkB expression and phosphorylation was observed as early as 30 min after 5-HT7 receptor activation. In addition to full-length TrkB, kinase domain-deficient forms may be expressed and act as dominant-negative proteins toward the full length receptor. We have identified distinct patterns of TrkB isoform expression across our cell lines and cortical cultures. Although TrkB receptor expression is regulated by cyclic AMP and Gαs-coupled GPCRs in several systems, we demonstrate that, depending on the model system, pathways downstream of both Gαs and Gα12 are involved in the regulation of TrkB expression by 5-HT7 receptors. Given the number of psychiatric and degenerative diseases associated with TrkB/BDNF deficiency and the current interest in developing 5-HT7 receptor ligands as pharmaceuticals, identifying signaling relationships between these two receptors will aid in our understanding of the potential therapeutic effects of 5-HT7 receptor ligands.

## Introduction

Neurotrophins, or neurotrophic factors, include nerve growth factor (NGF), brain derived neurotrophic factor (BDNF), neurotrophin-3 (NT-3) and neurotrophin-4/5 (NT-4/5) (Skaper, 2012). One of the receptors for BDNF is the tropomyosin-related kinase B (TrkB) receptor (Schecterson and Bothwell, 2010). TrkB receptors are transmembrane receptor tyrosine kinases (RTKs) found in dendritic spines, axons and neuronal cell bodies. There are three TrkB isoforms, full-length TrkB (TrkB-FL), TrkB-Shc, and TrkB-T1, that all have the same extracellular ligand-binding domain and transmembrane domain, however, the T-Shc and TrkB-T1 receptors are truncated and lack tyrosine kinase activity found in TrkB-FL (Fenner, 2012). Both TrkB-T1 and TrkB-T-Shc receptors are thought to modulate TrkB-FL activity by forming heterodimers with TrkB-FL receptors as well as by competing for and sequestering BDNF (Fenner, 2012; Gomes et al., 2012; Wong and Garner, 2012). TrkB-FL receptors can be phosphorylated at specific tyrosine residues including tyrosine 816 (Y816), the site for activating phospholipase Cγ (Huang and Reichardt, 2003). Recent work suggests that the relative number of TrkB isoforms expressed (e.g., TrkB-FL vs. TrkB-T1) is key for the neuroprotective signaling of BDNF. For example, increases in TrkB-T1 (via increased transcription, not as a degradation product of the full-length receptor) decrease TrkB-FL activity after excitotoxicity (Gomes et al., 2012).

A common theme in the regulation of TrkB signaling involves the second messenger cyclic AMP. BDNF-induced TrkB phosphorylation is regulated by cyclic AMP signaling in hippocampal neurons (Ji et al., 2005) and TrkB cell surface expression is regulated by cyclic AMP in retinal ganglion cells (RGCs) (Meyer-Franke et al., 1998). TrkB expression is also regulated by cyclic AMP signaling in several other systems (Heo et al., 2013). For example, the activation of the Gαs-coupled adenosine 2A receptors increases TrkB expression in the spinal cord (Golder et al., 2008). A2A receptors also promote an increased expression and release of the BDNF in rat cortical cultures (Jeon et al., 2011). In addition to changes in protein expression, GPCRs can increase RTK activation via a process called transactivation: adenosine 2A receptors and the Gαs-coupled dopamine D1 receptor are both able to transactivate TrkB (Lee et al., 2002; Wiese et al., 2007; Iwakura et al., 2008). 5-HT7 receptors are also reported to couple to Gαs (Bard et al., 1993; Lovenberg et al., 1993; Ruat et al., 1993) as well as Gα12 (Kvachnina et al., 2005).

TrkB-FL has been linked to neuroprotective effects after CNS insults by excitotoxicity, amyloid-β, and the HIV protein gp120 (Almeida et al., 2005; Nosheny et al., 2005; Kitiyanant et al., 2012) and changes in TrkB-FL are associated with neurodegenerative diseases (Longo et al., 2007; Zuccato and Cattaneo, 2009). In schizophrenia, as our understanding of the condition has shifted to viewing the disease as a neurodevelopmental illness, there has been an increased focus on neurotrophic factor signaling in its pathophysiology (Kalkman, 2009) and there is a strong association with BDNF with valine at position 66 (instead of methionine) and the illness (Rybakowski, 2008). Decreases in BDNF signaling are also associated with glaucoma and retinal degeneration (Gupta et al., 2014). A direct physiological link between 5-HT7 receptors and TrkB expression has been observed in a model of phrenic long-term facilitation (pLTF), a form of plasticity in the respiratory system that occurs as a response to hypoxia (Mitchell and Johnson, 2003). This centrally-mediated form of potentiation is associated with changes in 5-HT, BDNF, and TrkB signaling (Fuller et al., 2001; Baker-Herman et al., 2004). Recently, Hoffman and Mitchell demonstrated that spinal 5-HT7 receptor activation by the 5-HT7 receptor agonist, AS-19, was able to induce phrenic motor facilitation (PMF) that was dependent on new TrkB synthesis and activation (Hoffman and Mitchell, 2011). Initially these authors proposed a model whereby 5-HT7 receptor agonists might promote pLTF after hypoxia; however subsequent findings suggested the opposite: 5-HT7 agonists countered pLTF and antagonists (SB-269970) facilitated pLTF in conjunction with 5-HT2 receptor activation (Hoffman and Mitchell, 2013).

We have recently reported that long-term (24 h) application of 5-HT7 agonists (5-carboxamidotryptamine (5-CT) and LP 12) increases the expression of the platelet-derived growth factor (PDGF) β receptor in a PKA-dependent manner (Vasefi et al., 2012) and this increase in PDGF receptor expression was sufficient to protect primary hippocampal neurons against NMDA-induced excitotoxicity (Vasefi et al., 2013). Given that TrkB expression is regulated by several other Gαs-coupled receptors, we examined the ability of 5-HT7 agonists and antagonists to regulate TrkB receptor isoform expression and phosphorylation in primary mouse cerebral cortical cultures, the human neuroblastoma-derived SH-SY5Y cell line, and the RGC line 5 (RGC-5).

## Materials and methods

### Reagents and antibodies

LP12 [4-(2-Diphenyl)-*N*-(1,2,3,4-tetrahydronaphthalen-1-yl)-1-piperazinehexanamide hydrochloride] and H89 were purchased from Sigma-Aldrich (St. Louis, MO). SB 258719 ((R)-3,N-Dimethyl-N-[1-methyl-3-(4-methylpiperidin-1-yl)propyl]benzene sulfonamide) and SB 269970 (R-3-(2-(2-(4-methylpiperidin-1-yl)ethyl)-pyrrolidine-1-sulfonyl)-phenol were purchased from Tocris (Ellisville, MO, USA). Rhosin was purchased from Calbiochem (Billerica, MA). Antibodies against TrkB (catalog #sc-8316, used at a dilution of 1:500) and β-actin (catalog #sc-81178, used at a dilution of 1:500) were purchased from Santa Cruz (Santa Cruz, CA). The TrkB-Y816 antibody (catalog #ab75173, used at a dilution of 1:500) was purchased from Abcam (Toronto, ON). The secondary antibodies [horseradish peroxidase (HRP) enzyme-conjugated mouse polyclonal IgG and HRP enzyme conjugated rabbit polyclonal IgG] were both purchased from Thermo Fisher (Pittsburgh, PA).

### Primary cerebral cortical cultures

E17 to E19 mouse embryos were removed from pregnant CD-1 mice (Harlan, Indianapolis, IN) and transferred to chilled dissection media (15 mL HEPES buffer in 500 mL HBSS with 2.5 g glucose (0.6%), 10 g sucrose (2%), pH 7.4, final osmolality 320–335). The cortex was removed, separated, and trypsinized with 0.25% trypsin for 20 min at 37°C. Cells were then plated on poly-d-lysine-coated culture dished and grown at 37°C in a humidified atmosphere of 95% air and 5% CO_2_. Cells were plated with plating media (DMEM, supplemented with 10% fetal bovine serum, 10% horse serum) for the first 2–4 h until attached and then with feeding media consisting of Neurobasal medium and B-27 supplement (Life Technologies, Burlington, ON) without serum. Drug treatments were performed 7–10 days after plating the cells. Media was changed twice per week. To stop the overgrowth of non-neuronal cells, a mitotic inhibitor (81 μM 5-fluoro-2′-deoxyuridine and 200 μM uridine added to media) was added for 24 h once cells reached confluency.

### Cell culture

SH-SY5Y cells were grown on DMEM with Ham's F12 in a 1:1 ratio (Thermo Fisher), 10% fetal bovine serum and 1% penicillin/streptomycin. Media was changed every 2–3 days and cells were kept at 37°C and 5% CO_2_. Cells were serum and antibiotic deprived 24 h prior to drug treatment to prevent any interactions with growth factors. The RGC-5 cell line was grown on Dulbecco's Modified Eagle Medium (DMEM) with low glucose (Thermo Fisher), 10% horse serum and 1% penicillin/streptomycin. Media was changed every 2 days and cells were kept at 37°C and 5% CO_2_.

### Western blot

After drug treatment, cells were washed with phosphate-buffered saline (PBS) and lysed in chilled lysis buffer (20 mM Tris-HCl at pH 7.5, 150 mM NaCl, 1 mM EDTA, 1 mM EGTA, 30 mM sodium pyrophosphate, 1 mM β-glycerophosphate, 1 mM sodium orthovanadate, and 1% Triton X-100; supplemented with Halt Protease and Phosphatase Inhibitor (Thermo, Fisher, Markham, Ontario) prior to use). Cells were scraped, sheared using 26 gage needles, and centrifuged at 14,000 × g for 20 min at 4°C and the supernatant was collected. Protein concentrations were determined using a BCA protein assay protocol (Thermo Fisher). Homogenates were subjected to SDS-PAGE and proteins were transferred to nitrocellulose membranes, blocked with 5% non-fat dry milk in Tris-buffered saline and 0.1% Tween-20 for 1 h at room temperature or overnight at 4°C, and incubated in primary antibodies for 1 h at room temperature or overnight at 4°C. Membranes were washed three times in Tris-buffered saline with 0.1% Tween-20, incubated with HRP-conjugated secondary antibodies for 1 h at room temperature, washed again, and bound antibodies were visualized by enhanced chemiluminescence using Luminata Crescendo substrate (Millipore, Etobicoke, Ontario). Images of Western blots were taken on a Kodak 4000MM Pro Imaging Station, and densitometric analyses were performed using Kodak Molecular Imaging software. Protein bands were then identified by their molecular weights: TrkB-FL and TrkB-Y816 at 145 kDa, TrkB T1 at 95 kDa, TrkB T-Shc at 105 kDa, and bands were normalized to the loading control, β-actin.

### Statistical analysis

Mean protein expression/phosphorylation and standard errors were calculated using Microsoft Excel. GraphPad Prism® software was used for graphing and for analyzing statistical significance using a One-Way ANOVA with a Dunnett's or Bonferroni's post-test. Significance level was set to α = 0.05.

### Animals

All animal experiments were performed in agreement with the guidelines of the policies on the Use of Animals at the University of Waterloo under animal utilization project proposal (AUPP) #13-24 in accordance with standards of the Canadian Council on Animal Care.

## Results

### 24 h activation of 5-HT7 receptors increase TrkB expression

**Figure 1:**
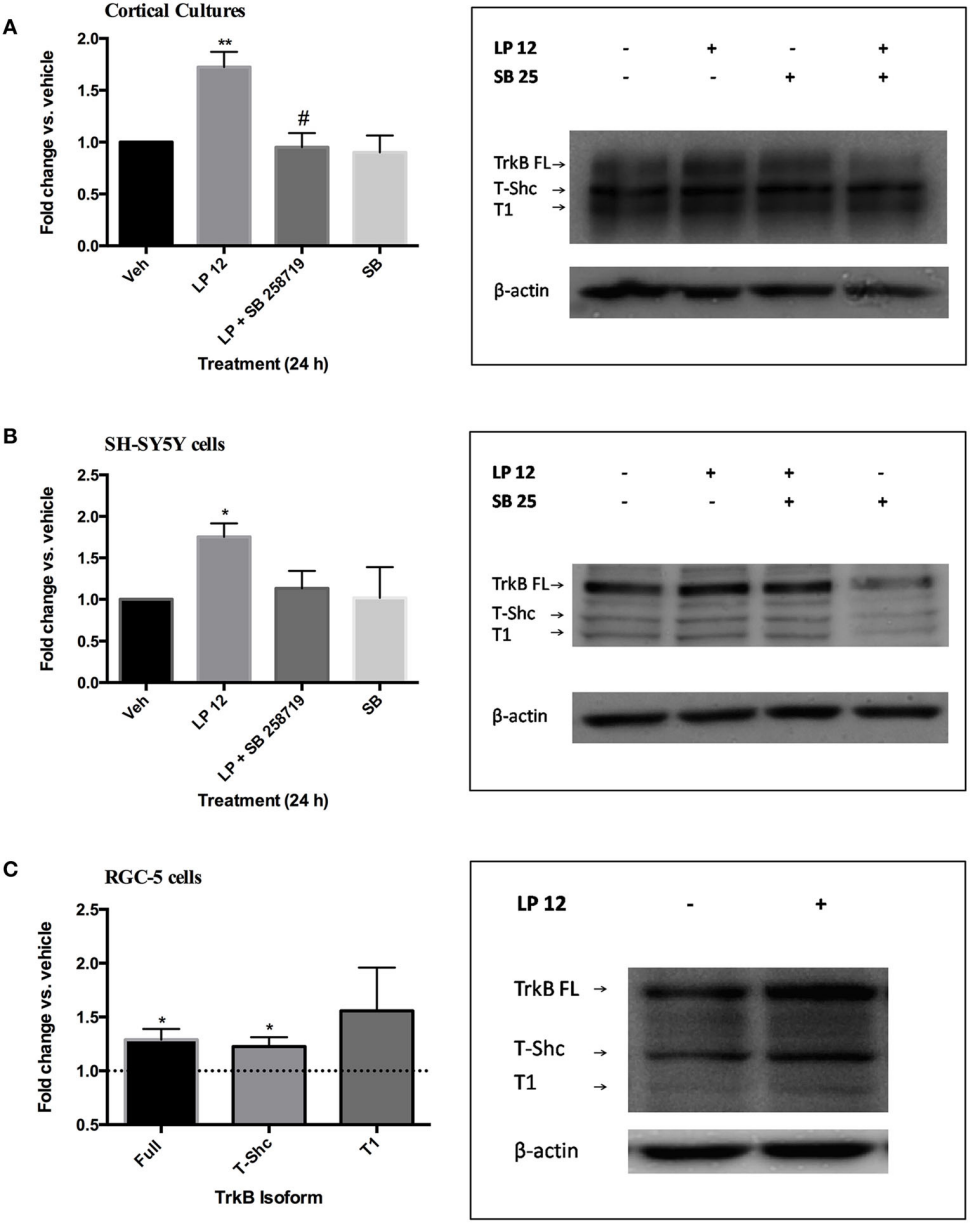
**5-HT7 receptor activation increases TrkB receptor expression in several model systems**. **(A)** Primary mouse cerebral cortical cultures were treated for 24 h with vehicle, 300 nM LP 12, 1 μM SB 258719, or both. The expression of TrkB-FL was normalized to β-actin and compared to the untreated sample (vehicle). ^**^p < 0.01 compared to vehicle, #, *p* < 0.05 compared to LP 12, *n* = 5, ANOVA analysis with Bonferroni's post-test. Representative western blots are shown for all TrkB isoforms and β-actin. Note that for “A” only the order of samples in the blot is different from the graph. **(B)** SH-SY5Y cells were treated as in “A.” ^*^p < 0.05, *n* = 10. **(C)** RGC-5 cells were treated with vehicle or 300 nM LP 12 for 24 h. The expression of TrkB-FL (145 kDa), TrkB-T-Shc (105 kDa) and TrkB-T1 (95 kDa) were normalized to β-actin and compared to vehicle. ^*^p < 0.05, *n* = 7–9, one-sample *t*-test. Representative blots are shown for all TrkB isoforms and β-actin.

Based on our previous findings that 24 h treatment of SH-SY5Y cells and primary cultures with 5-HT7 receptor agonists increased the expression of the PDGFβ receptor, we incubated primary mouse cerebral cortical cultures with the 5-HT7 receptor agonist LP 12 (300 nM) in the absence or presence of the 5-HT7 receptor antagonist SB 258719 (1 μM). LP 12 increased the expression of full-length TrkB (TrkB-FL) and this was blocked by the antagonist (Figure 1A). The same treatments resulted in similar effects in SH-SY5Y cells (Figure 1B). In both cell types, LP 12-induced increases in TrkB were also blocked using the inverse 5-HT7 receptor agonist, SB 269970 (data not shown). The concentration of 300 nM LP 12 was chosen based on our previous work on the regulation of PDGFβ receptor expression in similar model systems (Vasefi et al., 2013). Preliminary experiments in SH-SY5Y cells demonstrated that the application of concentrations higher than 300 nM resulted in sub-maximal increases in TrkB receptor expression (data not shown). In addition to TrkB-FL, we detected the expression of TrkB-T1 at 95 kDa and TrkB-T-Shc at 105 kDa. In both cortical neurons and SH-SY5Y cells, LP 12 treatment also resulted in an increase in TrkB-T-Shc and T1 by approximately 1.5 fold however these changes were not significant. In RGC-5 cells, 24 h LP 12 treatment significantly increased TrkB-FL and TrkB-T-Shc and there was a trend toward an even larger increase in TrkB-T1 but the result was much more variable and not statistically significant (Figure 1C). In all three model systems, the phosphorylation of Y816 on the full length TrkB receptor (a phosphorylation site associated with increased TrkB receptor activity; Huang and Reichardt, 2003) was significantly increased over baseline (1.40 ± 0.15, *n* = 7 in cortical neurons, 1.34 ± 0.12, *n* = 7 in SH-SY5Y cells, and 1.31 ± 0.12, *n* = 7 in RGC-5 cells).

### 2 h activation of 5-HT7 receptors increases TrkB expression

**Figure 2:**
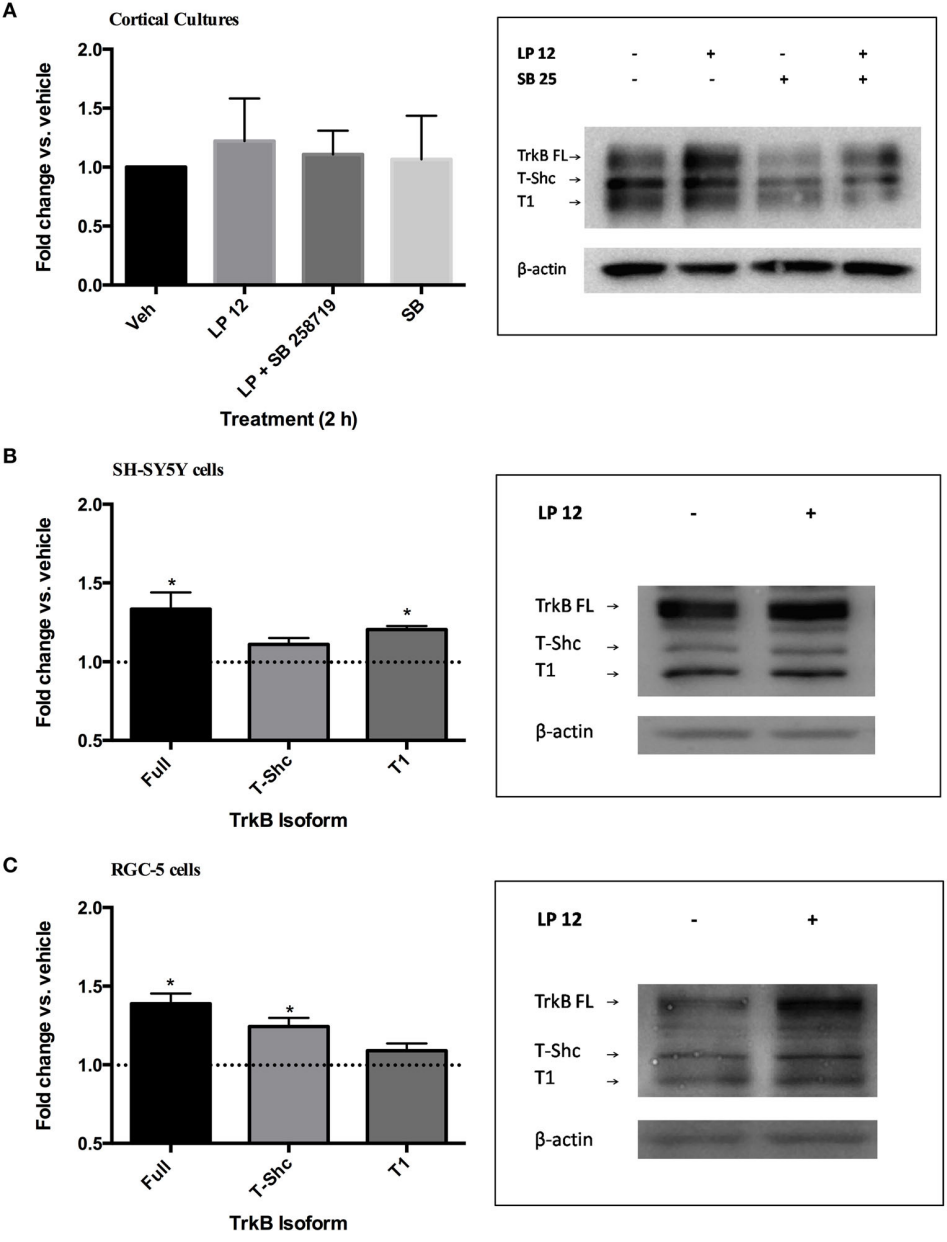
**Short-term incubation with LP 12 differentially alters TrkB isoform expression**. **(A)** Cortical cultures were treated as in Figure 1A for 2 h. The expression of TrkB-FL was normalized to β-actin and compared to the untreated sample (vehicle). *n* = 5. Representative blots are shown for each TrkB isoform and β-actin. **(B)** SH-SY5Y cells were treated for 2 h with 300 nM LP 12. Expression of TrkB-FL, T-Shc, and T1 were normalized to β-actin and compared to vehicle. ^*^p < 0.05, *n* = 3, one-sample *t*-test. Representative blots are shown for all TrkB isoforms and β-actin. **(C)** RGC-5 cells were treated as in “B.” *n* = 3–6.

We then investigated whether a shorter incubation of cortical neurons, SH-SY5Y or RGC-5 cells with LP 12 would result in similar changes in TrkB expression and phosphorylation. In cortical cultures, 2 h treatment with LP 12 failed to alter TrkB receptor expression (Figure 2A). Similarly, 2 h incubation of *ex vivo* hippocampal slices resulted in variable changes in TrkB expression, however this treatment significantly increased TrkB Y816 phosphorylation (1.24 ± 0.06 fold vs. vehicle, *n* = 3, *p* < 0.05, unpaired *t*-test, data not shown). In SH-SY5Y and RGC-5 cells, LP 12 treatment increased the expression of TrkB-FL but differentially increased TrkB-T1 in SH-SY5Y cells and T-Shc in RGC-5 cells (Figures 2B,C). A significant increase in Y816 phosphorylation was observed in RGC-5 cells (1.43 ± 0.09, *n* = 4) but not SH-SY5Y cells (1.22 ± 0.08, *n* = 6) and cortical neurons (1.59 ± 0.39, *n* = 5) after 2 h LP 12 treatment.

### Both Gαs and Gα12 pathways are involved in 5-HT7 receptor-induced TrkB expression

**Figure 3:**
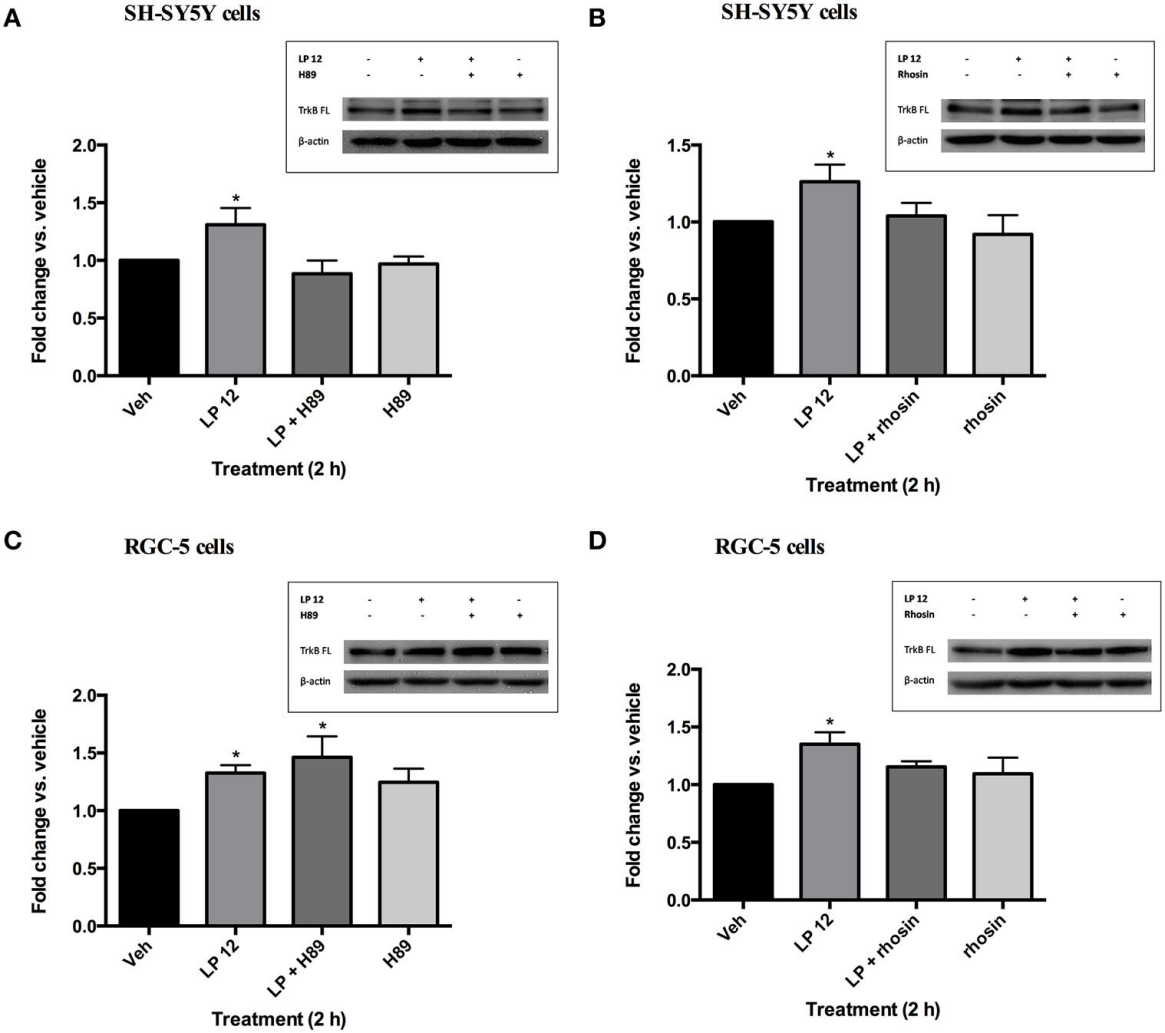
**Different pathways are involved in 5-HT7 receptor-induced increases in TrkB expression in SH-SY5Y cells and RGC-5 cells**. SH-SY5Y cells were pre-treated with 10 μM H89 **(A)** or 30 μM rhosin **(B)** for 30 min prior to the addition of 300 nM LP 12 for 2 additional h. RGC-5 cells were similarly treated with H89 **(C)** or rhosin **(D)** prior to incubation with LP 12. The expression of TrkB-FL was normalized to β-actin and compared to the untreated sample (vehicle). ^*^p < 0.05 compared to vehicle, *n* = 4 **(A–C)**, *n* = 5 **(D)**, ANOVA analysis with Dunnett's post-test.

There is evidence that 5-HT7 receptors couple to both Gαs and Gα12 (Bard et al., 1993; Lovenberg et al., 1993; Ruat et al., 1993; Kvachnina et al., 2005). To determine which G protein pathway(s) leads to an increase in TrkB expression we incubated SH-SY5Y and RGC-5 cells with the cyclic AMP-dependent protein kinase (PKA) inhibitor, H89, or the RhoA inhibitor, rhosin. In SH-SY5Y, pretreatment with H89 (30 min, 10 μM) attenuated the increase in TrkB (FL) by LP 12 (Figure 3A). However, the RhoA inhibitor, rhosin (30 min, 30 μM) also inhibited the increase in TrkB expression (Figure 3B). In contrast, rhosin, but not H89, blocked LP 12-induced increases in TrkB expression in RGC-5 cells (Figures 3C,D). Taken together these results suggest that multiple G protein pathways may be involved in promoting TrkB expression downstream of the 5-HT7 receptor and that different pathways may be involved in different systems.

### LP 12 treatment results in different patterns of TrkB isoform expression in SH-SY5Y and RGC-5 cells

**Figure 4:**
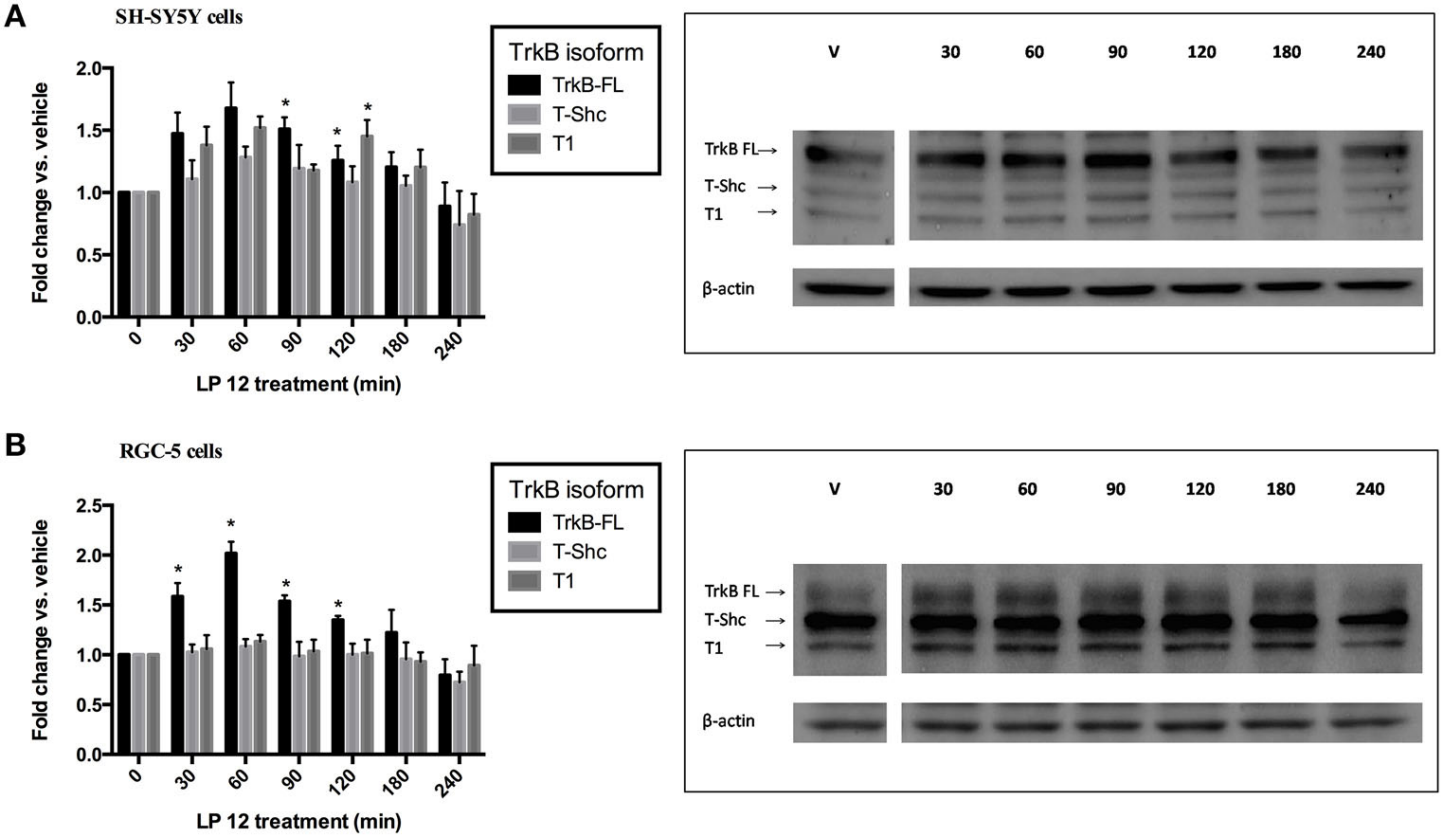
**Time course for the effects of LP 12 on TrkB isoform expression**. SH-SY5Y cells **(A)** or RGC-5 cells **(B)** were incubated for 30–240 min with 300 nM LP 12. Expression of TrkB-FL, T-Shc, and T1 were normalized to β-actin and compared to vehicle. ^*^p < 0.05, *n* = 3–4 (SH-SY5Y), *n* = 5–6 (RGC-5 cells), ANOVA with Dunnett's post-test. Representative blots are shown for all TrkB isoforms and β-actin.

We performed a time course experiment with LP 12 in the two models, SH-SY5Y and RGC-5 cells, that displayed differences in TrkB expression at 2 h. Both cell types were treated for 0.5, 1, 1.5, 2, 3, or 4 h with 300 nM LP 12 and changes in the three TrkB receptor isoforms were measured. As shown in Figures 4A,B, we observed very rapid changes in TrkB isoform expression as well as different patterns of TrkB isoform expression. In SH-SY5Y cells, changes were observed with all isoforms (however only changes in TrkB-FL at 1.5 and 2 h and TrkB-T1 at 2 h were significant, Figure 4A). In RGC-5 cells, changes in TrkB-FL were observed at earlier time points and LP 12 treatment did not significantly affect the expression of the other isoforms (Figure 4B). Note that unlike Figure 2C, in these five time-course experiments we did not observe changes in TrkB-T-Shc levels.

### 5-HT7 receptor-induced TrkB receptor transactivation

**Figure 5:**
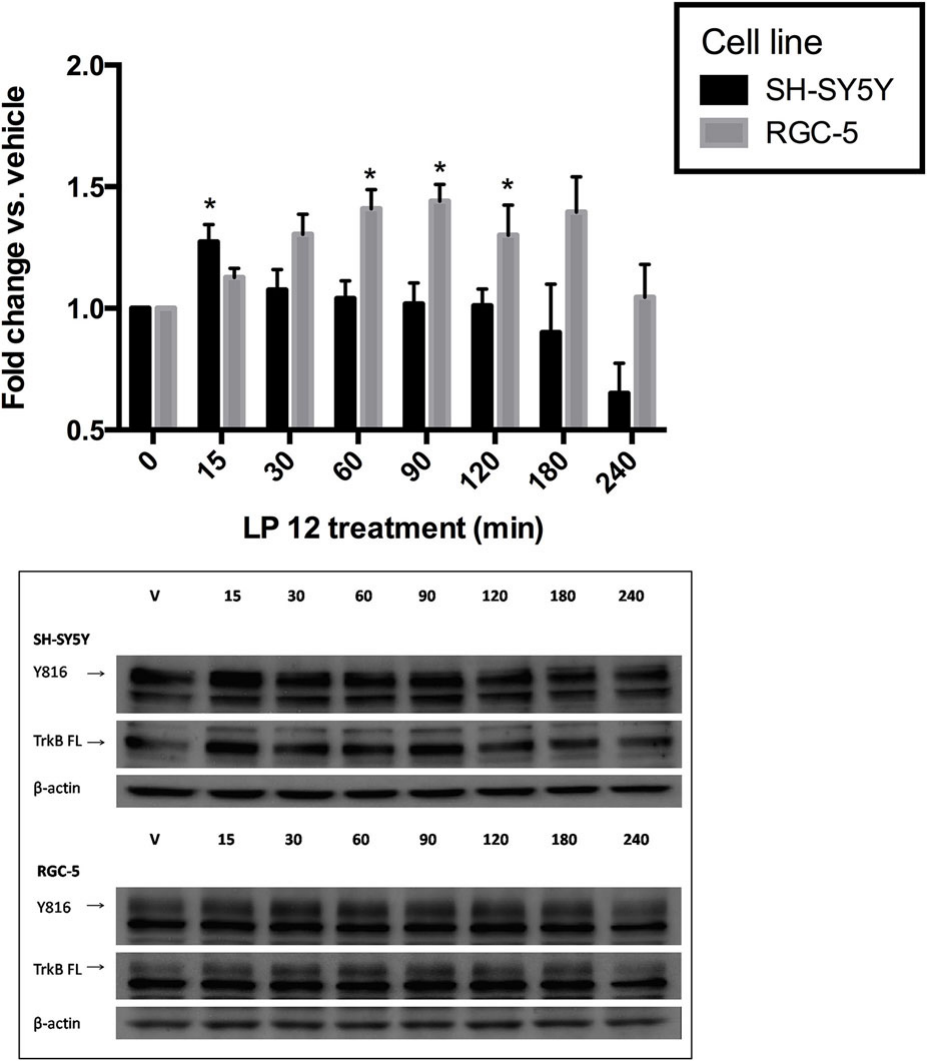
**5-HT7 receptor transactivation of the TrkB receptor**. SH-SY5Y cells (black bars, upper three blots) or RGC-5 cells (gray bars, lower three blots) were incubated for 15–240 min with 300 nM LP 12. Phosphorylation of the TrkB was measured using an anti-TrkB-phospho-Y816 antibody and compared to vehicle. ^*^p < 0.05, *n* = 4 (SH-SY5Y), *n* = 5 (RGC-5 cells), ANOVA analysis with Dunnett's post-test. The data from each cell line were collected independently thus the statistical test for each cell line were analyzed independently, however the data are presented together for comparison purposes. Representative blots are shown for phosphor-Y816, TrkB isoforms, and β-actin.

In addition to being activated by its ligand, BDNF, TrkB receptors can be transactivated after activation of GPCRs including adenosine 2A and dopamine D1 receptors (Lee et al., 2002; Wiese et al., 2007; Iwakura et al., 2008). To determine if 5-HT7 receptors are able to acutely transactivate TrkB receptors we performed a time-course similar to that described in Figure 4 but with the addition of a 15 min time point. Interestingly, in SH-SY5Y cells, TrkB receptor transactivation was very brief (15 min) whereas in RGC-5 cells, the transactivation time course was more typical of that reported by others; the increase in TrkB receptor phosphorylation after LP 12 treatment lasted up to 2 h (Figure 5).

## Discussion

In cortical cultures, SH-SY5Y cells, and RGC-5 cells, 24 h LP 12 treatment increased the expression of full length TrkB expression and its basal phosphorylation at Y816 and although the expression of T-Shc and T1 were also elevated over baseline in all models, only TrkB-T-Shc was significantly increased in RGC-5 cells. Over a shorter time period, differences in 5-HT7 receptor regulation of TrkB receptor isoforms were observed. In SH-SY5Y cells, 2 h LP 12 treatment increased TrkB-FL and TrkB-T1 isoforms, whereas TrkB-FL and TrkB-T-Shc were increased in RGC-5 cells. TrkB-T1 and T-Shc are generally ascribed dominant-negative activity toward full length TrkB (Gomes et al., 2012; Wong and Garner, 2012) as well as regulating TrkB cell surface expression (Haapasalo et al., 2002). Thus, signaling pathways that regulate TrkB expression and/or cell surface localization will produce a complex net effect of changes in TrkB-FL and the dominant negative forms. In neurons, this complexity is compounded by the relative localization of TrkB forms at synapses, cell bodies, etc. Although we observed changes in TrkB isoform expression in cortical cultures at 24 h, we did not see reliable changes over shorter time periods. This may be due to the reduced sensitivity of measuring changes in mixed neuronal cultures or the relatively lower expression of 5-HT7 receptors in the cortex compared to other brain regions (Horisawa et al., 2013).

Based on our previous work (Vasefi et al., 2012), and the literature identifying Gαs-coupled receptors and cyclic AMP pathways in regulating TrkB receptor expression (Ji et al., 2005; Heo et al., 2013), we predicted that 5-HT7 receptor activation would increase TrkB receptor levels via the Gαs-cyclic AMP-PKA pathway. However, 5-HT7 receptors are also reported to couple to Gα12 and Rho GTPase pathways, specifically the activation of RhoA and cdc42 (Kvachnina et al., 2005). As both of these monomeric G proteins are involved in cell growth in neurons, the Ponimaskin group recently demonstrated the involvement of 5-HT7 receptor/Gα12 in dendritic growth and synaptic activity/plasticity (Kobe et al., 2012). In our model systems, the changes in TrkB expression involved PKA and RhoA signaling pathways, or both, depending on the cell line. Many of the effects of 5-HT7 receptor signaling observed by Kobe et al. were in developing neurons after a 4 days treatment with the 5-HT7 (and 5-HT1a) agonist, 5-CT (Kobe et al., 2012). We observed changes in TrkB expression after 24 h as well as over shorter time periods. Interestingly, the TrkB receptor and BDNF signaling are intimately involved with many of the processes Kobe et al. observed in their systems (Kobe et al., 2012): see recent reviews on the involvement of TrkB/BDNF in dendritic spine formation (Bennett and Lagopoulos, 2014), synaptogenesis (Luikart and Parada, 2006), and synaptic plasticity (Lu et al., 2014).

The term transactivation is used to describe acute and usually very brief (5–15 min) GPCR-induced activation of RTKs that does not involve changes in RTK protein expression. This may occur through entirely intracellular signal transduction pathways, as in the case of 5-HT-induced transactivation of the PDGFβ and TrkB receptor in SH-SY5Y cells (Kruk et al., 2013). Another mechanism involves a GPCR-induced metalloproteinase-dependent shedding of growth factor ligand (such as EGF) to activate its RTK (Wetzker and Bohmer, 2003). TrkB receptor transactivation often differs from the transactivation of other RTKs in that it typically manifests as a longer-lasting increase in receptor phosphorylation (Lee et al., 2002). Interestingly, we observed three distinct patterns of TrkB receptor transactivation by LP 12 in the three systems we investigated. Transactivation of TrkB was not reliably observed in cortical cultures. In SH-SY5Y, we observed a brief (15 min) transactivation that quickly returned to baseline, typical of many other RTK transactivation pathways. In RGC-5 we observed a transactivation typical of the TrkB receptor: one that was delayed in onset but much longer lasting. It remains to be determined what factors contribute to TrkB transactivation differing kinetically from the transactivation of other RTKs.

Given the interest in targeting growth and neurotrophic factor signaling pathways in CNS disease, an attempt to develop direct small molecule modulators for RTKs are ongoing, and in fact the TrkB receptor has been at the forefront of this avenue of investigation (Longo et al., 2007; Webster and Pirrung, 2008). An alternative approach to modulating RTKs in the CNS is to identify GPCRs linked to RTK signaling and expression. For 5-HT7 receptors, we have demonstrated such an approach *in vitro*: the 5-HT7 receptor ligand, LP 12, increases PDGFβ receptor expression in hippocampal neurons to provide neuroprotection against excitotoxicity (Vasefi et al., 2013). The development of selective 5-HT7 receptors able to cross the blood-brain barrier (Monti et al., 2014) will allow us to test the ability of 5-HT7 receptor ligands to modify RTK activity *in situ* in the CNS. Glaucoma is a family of eye diseases that results in visual field loss and eventual blindness (Weinreb et al., 2014). A reduction in BDNF is thought to contribute to the complex mechanisms of RGC death in glaucoma (Gupta et al., 2014). In rat, application of exogenous BDNF to the retina through intravitreal injection resulted in a promotion of RGC survival (Ko et al., 2001) thus there is extensive interest in delivering exogenous BDNF to the retina as a glaucoma therapy. This has resulted in several approaches directed toward the delivery of neuroprotective genes to the retina in a less-invasive manner (Park et al., 2012; Alqawlaq et al., 2014). An alternative approach would be to exploit the 5-HT7 receptor's ability to promote TrkB expression and phosphorylation *in situ* in the retina. We began to explore this possibility *in vitro* using the RGC-5 cell line. Unfortunately, the integrity of the RGC-5 cell line has recently been compromised given investigations that revealed that the cell line does not express ubiquitous markers of RGCs and that its origin is from mouse and not rat as previously reported (Van Bergen et al., 2009). Nevertheless, 5-HT7-TrkB receptor linkages have been demonstrated here in two cell lines as well as in cortical cultures, and is likely present in the spinal cord (Hoffman and Mitchell, 2011, 2013). Therefore, further investigation of the possibility of 5-HT7 receptor-induced regulation in TrkB signaling in the retina and throughout the CNS is warranted.

## Author contributions

Anshula Samarajeewa contributed most of the SH-SY5Y and RGC-5 data. Lolita Goldemann contributed much of the 24 h data in SH-SY5Y cells. Maryam S. Vasefi mentored several of the contributors as well as being involved in data collection. Nawaz Ahmed and Nyasha Gondora produced the data from cortical neurons. Chandni Khanderia collected RGC-5 data. John Mielke is the co-supervisor for Nyasha Gondora. Michael A. Beazely is the supervisor for undergraduate students Anshula Samarajeewa, Lolita Goldemann, and Chandni Khanderia as well as graduate students Maryam S. Vasefi, Nawaz Ahmed, and Nyasha Gondora. Anshula Samarajeewa, Maryam S. Vasefi, Nawaz Ahmed, Nyasha Gondora, and Michael A. Beazely wrote and edited the manuscript.

### Conflict of interest statement

The authors declare that the research was conducted in the absence of any commercial or financial relationships that could be construed as a potential conflict of interest.

## Abbreviations

5-HT: serotonin
BDNF: brain-derived neurotrophic factor
cyclic AMP: cyclic adenosine mono-phosphate
GPCR: G protein-coupled receptor
PDGF: platelet-derived growth factor
pLTF: phrenic long-term facilitation
PMF: phrenic motor facilitation
RGC: retinal ganglion cell
RTK: receptor tyrosine kinase
TrkB: tropomyosin-related kinase B.
